# Techniques for detecting chromosomal aberrations in myelodysplastic syndromes

**DOI:** 10.18632/oncotarget.17698

**Published:** 2017-05-09

**Authors:** Qibin Song, Min Peng, Yuxin Chu, Shiang Huang

**Affiliations:** ^1^ Cancer Center, Renmin Hospital of Wuhan University, Wuhan, China; ^2^ Molecular department, Kindstar Global, Wuhan, China

**Keywords:** chromosomal aberration, technique, myelodysplastic syndromes

## Abstract

Myelodysplastic syndromes (MDS) are a group of heterogeneous hematologic diseases. Chromosomal aberrations are important for the initiation, development, and progression of MDS. Detection of chromosomal abnormalities in MDS is important for categorization, risk stratification, therapeutic selection, and prognosis evaluation of the disease. Recent progress of multiple techniques has brought powerful molecular cytogenetic information to reveal copy number variation, uniparental disomy, and complex chromosomal aberrations in MDS. In this review, we will introduce some common chromosomal aberrations in MDS and their clinical significance. Then we will explain the application, advantages, and limitations of different techniques for detecting chromosomal abnormalities in MDS. The information in this review may be helpful for clinicians to select appropriate methods in patient-related decision making.

## INTRODUCTION

Myelodysplastic syndromes (MDS) are a group of heterogeneous hematopoietic stem cell (HSC) disease, characterized by ineffective hematopoiesis, a variable degree of peripheral cytopenia, hypercellular bone marrow with morphologically defined dysplasia of cell lineages, and an increased propensity of evolving to acute myeloid leukemia (AML) [1, 2]. MDS often affects the elderly male patients with ages over 70 years. The incidence of MDS is reckoned about 3-5/100000 persons [3]. This incidence is predicted to escalate remarkably with an increase of ages [4, 5]. MDS comprises several different subtypes, including refractory anemia (RA), refractory anemia with ringed sideroblasts (RARS), refractory cytopenia with multilineage dysplasia (RCMD), refractory cytopenia with multilineage dysplasia and ringed sideroblasts (RCMD-RS), refractory anemia with excess blasts (RAEB), myelodysplastic syndrome unclassified (MDS-U), MDS associated with isolated del(5q) [6, 7].

It has been reported that approximately 70% of MDS patients have clonal chromosomal aberrations at initial diagnosis [8]. These chromosomal abnormalities have a great influence on the behavior of malignant cells, disease evolution, response to therapeutic drugs, and overall survival of MDS patients [9, 10]. A large variety of different chromosomal abnormalities have been depicted in MDS, such as loss or gain of chromosomal fragments, acquired uniparental disomy (UPD), and complex karyotypes [11]. Chromosomal loss may engender deletion of tumor suppressor genes (TSGs). Alternatively, chromosomal gain may activate oncogenes [12]. UPD defined as two copies of a chromosomal pair originate from one single parent during meiosis, may also increase genomic instability by activating oncogenes or inactivating TSGs in MDS [13]. Moreover, complex karyotype which is defined as the existence of ≥ 3 chromosomal aberrations usually contains both numerical and structural alterations [14]. Complex karyotype often implies an increased risk of progressing to AML and unfavorable outcomes in MDS patients [15]. These chromosomal aberrations appear to be mechanisms to interpret disease progression. Additionally, environmental risk factors may also engender the pathogenesis of MDS. For instance, iron overload-induced oxidative stress may inhibit hematopoiesis by altering the supportive bone marrow stroma environment [5]. Epigenetic alterations such as TET2 on chromosome 4q24 and IDH2 mutation on 15q26.1 for mitochondrial dysfunction also contribute to the pathogenesis of MDS [2]. Thus, detection of chromosomal abnormalities may afford valuable information for accurate diagnosis of MDS, and may also optimize current therapeutic strategies for MDS patients.

During the past several decades, a series of techniques have been developed for detecting chromosomal aberrations in MDS, including metaphase cytogenetics (MC), fluorescence *in situ* hybridization (FISH), spectral karyotyping (SKY), single nucleotide polymorphism arrays (SNP-A) genotyping, array-based comparative genomic hybridization (a-CGH), and targeted DNA sequencing. The advent of these techniques has contributed to the investigation of chromosomal changes in MDS, including unbalanced chromosomal deletions and gains as well as balanced translocations [16]. The chromosomal findings will enhance our understanding of the pathogenesis of MDS.

In this review, we will not only recapitulate the current knowledge of common chromosomal aberrations in MDS, but also summarize the techniques for detecting chromosomal aberrations in MDS. Specifically, we will also introduce the application, advantages and limitations of each technique.

## COMMON CHROMOSOMAL ABERRATIONS IN MDS

Del(5q), trisomy 8, del(20q), del(7q), monosomy 7, and complex karyotypes are the commonest chromosomal aberrations in MDS [17]. Loss, gain, and UPD of genomic materials in these chromosomes are associated with the initiation and progression of MDS. So we focus on reviewing these common chromosomal aberrations in MDS (Table 1).

**Table 1:** Chromosomal aberrations in MDS

Aberration type	Position	Significance	Reference
del(5q)	5q31	AML evolution	21
trisomy 8	cT8M	intermediate-risk	38,39
del(20q)	20q11.2- q13.1	exacerbate malignancy	46,47
del (7q)	7q22, 7q34	contribute to hematopoietic aberration	56
monosomy 7	-7	higher-risk, poor prognosis	66–68
complex karyotype	multiple	unfavorable outcome	76

### Deletion 5q

Heterozygous, interstitial deletions of chromosome 5q (del(5q)) are the commonest cytogenetic aberration in MDS [18], which accounts for approximately 30% of MDS subtypes [19]. MDS patients with isolated del(5q) often have a good prognosis, however, when accompanied with additional chromosomal aberrations, their prognosis becomes unfavorable [20]. The chromosome band 5q31 is the most frequently deleted region, including 2 different commonly deleted regions (CDRs). The proximal 5q31.1-q31.2 region is putatively related to an increased risk of evolving to AML [21]. Another distal CDR located in the 5q32-q33 bands is considered to involve the pathogenesis of 5q− syndrome and often prefigures a favorable prognosis [22]. Haploinsufficiency of many candidate genes may potentially alter hematopoiesis, resulting in the phenotype of MDS patients with del(5q) and malignant transformation [23]. For instance, RPS14 gene encodes a ribosomal protein small subunit 14 which influences the maturation of erythroid progenitor cells [24, 25]. Haploinsufficiency of RPS14 gene may affect the p53 pathway, and the subsequent loss of p53 rescues erythropoiesis and contributes to clonal progression [26]. Pathogenetic mechanisms in del(5q) MDS seem to involve hemizygous mutations in addition to haploinsufficiency, and may be modified by other somatic alterations influencing genes on other chromosomes [27]. Moreover, selection of particular treatment may rely on the presence of specific chromosomal aberrations. Low-risk, transfusion-dependent MDS patients with del(5q) are reported to respond well to lenalidomide [28–30]. So accurate detection of del(5q) is not only important for precise diagnosis of MDS, but also vital for individualized treatment of MDS patients.

### Trisomy 8

Trisomy 8 (+8) is one of the most frequent chromosomal gains in adult MDS patients [31], which accounts for 5% of all MDS patients in Western countries [32] and roughly 30-35% in Chinese MDS patients [33, 34]. According to the new revised IPSS (IPSS-R), isolated trisomy 8 in MDS is classified as intermediate cytogenetic risk group and should be considered with adequate evidence to diagnose MDS in patients with hypercellular or normal bone marrow [35]. Despite the association between particular chromosomal lesions and somatic mutations has not been clarified, several studies have reported that trisomy 8 was related to an IDH or ASXL1 mutation in MDS harboring trisomy 8 [36, 37].

Trisomy 8 (+8) can also be identified as a constitutional mosaicism (cT8M). A study have analyzed the existence of +8 in CD3+ lymphocytes and granulocytes from peripheral blood, as well as in oral mucosa cells from MDS patients with +8, in order to elucidate the incidence of cT8M in MDS and provide an accurate diagnostic and prognostic value for isolated +8. Cytogenetic analysis of peripheral blood found trisomy 8 in 5% to 65% of cells. FISH analysis also revealed trisomy 8 in 3% to 74% of granulocytes from all patients studied [38]. Complexity of chromosomal aberrations have a great impact on the overall survival (OS) of MDS patients. Those patients with isolated trisomy 8 have a median OS from 11 to 25 months, while patients with bone marrow blasts ≥ 5% combining trisomy 8 have relatively shorter OS and increased AML transformation [39].

Clonal heterogeneity has been regarded as a specific cytogenetic characteristic of MDS. Trisomy 8 may “come and go” as an independent clone or a single cell aberration. Usually, clonal evolution is a predictor for disease progression [3]. MDS patients with trisomy 8 and del(5q) as independent clone had a remarkably longer time to progress to AML than those with clonal evolution [40]. Analysis of whole gene expression revealed that most genes on chromosome 8 are overexpressed in AML trisomy 8. Hence the gene-dose effect may lead to leukemic progression of MDS with trisomy 8 [41]. Furthermore, MDS patients with trisomy 8 are more likely to respond to immunosuppressive agents than other subtypes of MDS [42].

### Deletion 20q

An interstitial deletion of chromosome 20q (del(20q)), is also prevalent in MDS, accounting for 3–7% of all MDS patients [43, 44]. Isolated del(20q) has been found both in primary and therapy-related MDS patients. Those patients often manifest anemia and thrombocytopenia, which involve bone marrow dysplasia [45]. Del(20q) is considered to derive from a pluripotent stem cell and may exacerbate malignancy due to the deletion of tumor suppressor genes [46]. In the past several years, many studies have been initiated to detect the CDR on chromosome 20. The CDR can be narrowed on chromosomal bands from 20q11.2 to 20q13.1, with variable sizes from 2.6 to 10.4 Mb [47]. These CDRs often subsume several key genes that may affect the pathogenesis and course of MDS. For example, the E2F1 gene on band 20q11.2 encodes a transcription factor, which involves in cell cycle control, proliferation modulation and p53-mediated apoptosis. Increased levels and activity of E2F1 transcription factor have been observed in myelodysplastic bone marrow [48, 49]. Isolated del(20q) in MDS is a favorable recurrent chromosomal aberration, with higher reticulocyte counts, fewer bone marrow blasts, and an indolent clinical course [50, 51]. The survival of patients with a del(20q) was considered to be significantly longer than other MDS patients [52]. Thus, MDS patients with isolated del(20q) usually have a relatively favorable prognosis.

However, as the size of chromosome 20 is too small, the traditional cytogenetic analysis is difficult to pinpoint chromosomal regions for its deletion [53]. So MDS with del(20q) may be further stratified by additional cytogenetic and molecular techniques.

### Deletion 7q

Deletion of chromosome 7q (del (7q)) is also frequently found in MDS and are associated with a poor prognosis [54]. The percentage of del(7q) cells is significantly higher in HSC and progenitor compartments than in lymphocytes of MDS patients [55]. Multiple investigations of MDS samples with interstitial del (7q) have identified 3 potential CDRs at chromosome bands 7q22, 7q34, and 7q35-36 [55]. Specifically, deletion of 7q22 in bone marrow cells could contribute to hematopoietic abnormalities, such as vandalized lymphoid repopulating potential, myeloid output discrepancy, and a remarkable proliferation of HSC [56]. Del (7q) may engender the haploinsufficiency of several critical genes implicated in hematological malignancies, subsuming MLL3, CUX1, and EZH2 [57–59], which are responsible for the leukemic progression of MDS [60]. Furthermore, UPD 7q and homozygous EZH2 mutation have been found in 10% of MDS patients. These chromosomal abnormalities often portend clonal evolution and highlight the vital role of del (7q) in the pathogenesis of MDS [61].

### Monosomy 7

Monosomal karyotype (MK) is defined as the existence of a single autosomal monosomy related with at least one additional structural alteration in the same clone, or at least 2 autosomal monosomies [62]. Monosomy 7 is the most prevalent chromosomal abnormality of MDS in childhood, and often exists as the sole cytogenetically visible chromosomal aberration [63, 64]. Immunophenotypic analysis of immature stem and progenitor cell compartments from patients with monosomy 7, showed expansion and dominance of the malignant –7 clone in granulocyte, macrophage progenitors, and other CD45RA+ progenitor compartments [65]. The monosomy 7 clone had a relative disadvantage in erythroid differentiation [65].

Monosomy 7 has been regarded as an independent predictor of survival in patients with higher-risk MDS. The addition of MK as a binary variable could improve the predictive accuracy of current models to estimate the survival of patients with MDS [66]. For example, a recent study has retrospectively analyzed 2080 primary patients, in order to elucidate the prognostic significance of MK in Chinese MDS patients. They have found that MK was significantly related to elderly patients, higher bone marrow blasts and relatively unfavorable cytogenetics. Monosomies of chromosome 5/7 were significantly associated with shorter OS by multivariate analysis [67]. Another study has investigated if an MK is related to OS independent of the number of cytogenetic aberrations in a population-based MDS cohort. They have found that monosomy 7 was responsible for worse OS in the entire cohort (median 6 vs 39 months), including those with a coexisting complex karyotypes (6 vs 17 months) [68]. Thus, MK predicts inferior survival of complex karyotypes in MDS patients.

Isolated monosomy 7 or monosomy 7 plus one additional aberration is associated with a median survival of 14.0 months and thus with an intermediate risk [19]. Consequently, early stem cell transplantation is recommended as soon as a monosomy 7 clone was detected [69].

Monosomy 7 is also the commonest chromosome abnormality in the course of evolution from MDS to AML in patients with different bone marrow failure syndromes and DNA repair deficiencies [70]. Thus, there are underlying aberrations leading to these constitutional disorders that also predispose to MDS and AML.

### Complex karyotype

Complex karyotype (CK) was defined as the existence of at least three chromosomal alterations and was especially prevalent in secondary MDS [71, 72]. Complex karyotypes and large number of chromosomal abnormalities may reflect an inherent chromosomal instability that contributes to disease progression. A higher incidence of complex karyotypes represents more aggressive disease [73]. The pathogenic mechanisms leading to complex karyotypes in MDS still remain vague. UPD may contribute to genomic instability by activating oncogenes and inactivating tumor suppressor genes, facilitating the development and progression of complex chromosomal aberrations [74]. Moreover, complex karyotypes in MDS may arise from gradual acquisition of genetic changes in individual cells during clonal evolution or by extensive chromosome fragmentation and reorganization at a single event known as chromothripsis [75].

Patients with complex karyotypes often imply an unfavorable outcome, a shorter median OS, only 3 months, and propensity toward malignant progression. Multiple chromosomal aberrations often portend an adverse prognosis and difficult treatment [76].

Analysis of complex karyotypes facilitates the identification of latent unbalanced chromosomal alterations and candidate regions of genes responsible for the progression of MDS. These regions can then be investigated further at the molecular level, which may render more accurate diagnosis of MDS and help to find potential targets for therapeutic interventions in the future.

## TECHNIQUES FOR DETECTING CHROMOSOMAL ABERRATIONS IN MDS

Cytogenetic findings are important for the diagnosis, prognosis evaluation and treatment selection of MDS patients [77]. Many techniques have been developed to detect chromosomal aberrations in MDS, such as metaphase cytogenetic (MC) analysis, FISH, Array-CGH, SNP-Array, SKY, and NGS (Table 2). Although these techniques are varying in depth, scope and cost, they are important for detecting diverse chromosomal abnormalities in MDS.

**Table 2:** Different techniques for detecting chromosomal aberrations in MDS

Technique	Application	Advantage	Shortcoming	Price
MC	visible chromosomal aberrations	Simple, whole chromosomal view	Low resolution, Can’t detect UPD	800 rmb
FISH	small and hidden chromosomal aberrations	Not rely on proliferating cells, High sensitivity	only detect particular chromosomal aberrations	2000 rmb
SKY	Unknown and complex chromosomal aberrations	display better pictures of karyotypes	Can’t detect structural aberration, low resolution	3500 rmb
SNP-A	Cryptic and complex chromosomal aberrations	high-resolution, can detect UPD	Can’t detect balanced translocation and inversion	5000 rmb
Array-CGH	Detect CNV and UPD	genome-wide analysis high-resolution	Can’t detect balanced rearrangements, low-level mosaicism and polyploidy	4500 rmb
Sequencing	CNV and structural variants, unknown mutation or aberrations	genome-wide analysis improved sensitivity monitor clonal mutations	Expensive, time-consuming, complicated bioinformatic analysis	6000 rmb

### Metaphase cytogenetics

**Figure 1:**
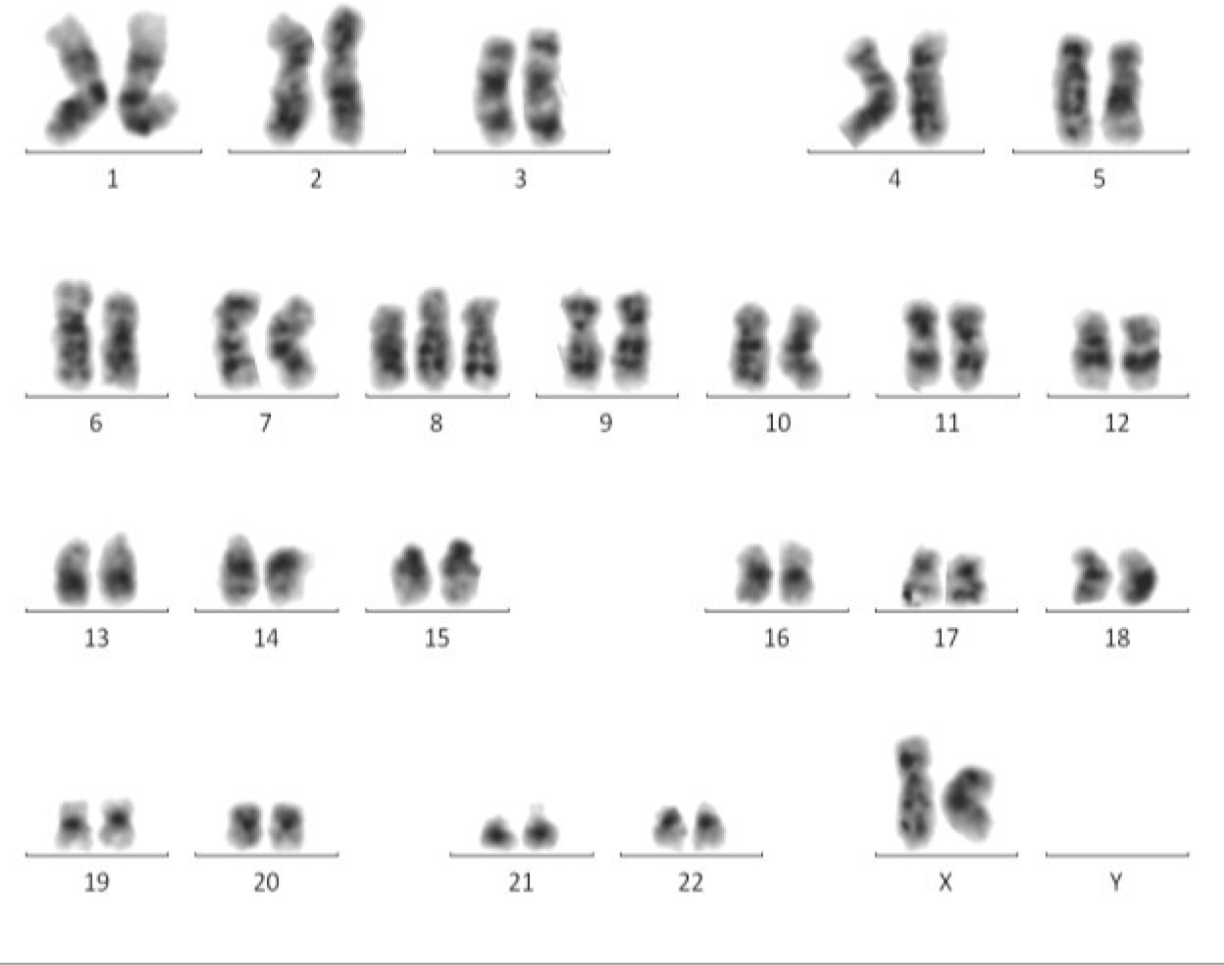
MC displays a whole chromosomal view of visible aberrations in a MDS patient Trisomy 8 is distinctly revealed by MC.

Metaphase Cytogenetics (MC) still remains the gold standard for detection of chromosomal aberrations in MDS [78]. This method can’t only identify unbalanced chromosomal lesions, including loss, gain, and trisomy (Figure 1), but also detect balanced chromosomal defects, such as translocation and inversion [79]. It can provide a whole chromosomal view of visible aberrations in chromosome number and structure simultaneously [80]. Furthermore, the simplicity of MC assay allows for the feasibility of discerning single cellular clones [81]. The chromosomal aberrations detected by MC often have strong prognostic value. These are the major advantages of MC.

However, about 40–50% of MDS patients don’t exhibit karyotype aberrations assessed by standard MC [82, 83]. The resolution of conventional MC is quite low. This method also requires proliferating cells, and to a large extent, relies on specialist experience for discriminating meaningful data [84]. As a result, traditional MC should be initiated by cytogenetic labs with rich experience in MDS.

Furthermore, even if 50% of MDS patients with abnormal karyotypes are identified by MC, it still can’t completely eliminate the existence of some cryptic chromosomal defects which often evades the detection by MC with relatively low resolution [85]. Most importantly, MC is unable to identify UPD because the chromosome banding patterns remain unaltered [86]. In some patients, MC may even fail to come up with informative results due to low resolution and non-dividing cells. Consequently, the technical limitations of MC may lead to underestimate of the extent of chromosomal abnormalities.

### FISH

**Figure 2:**
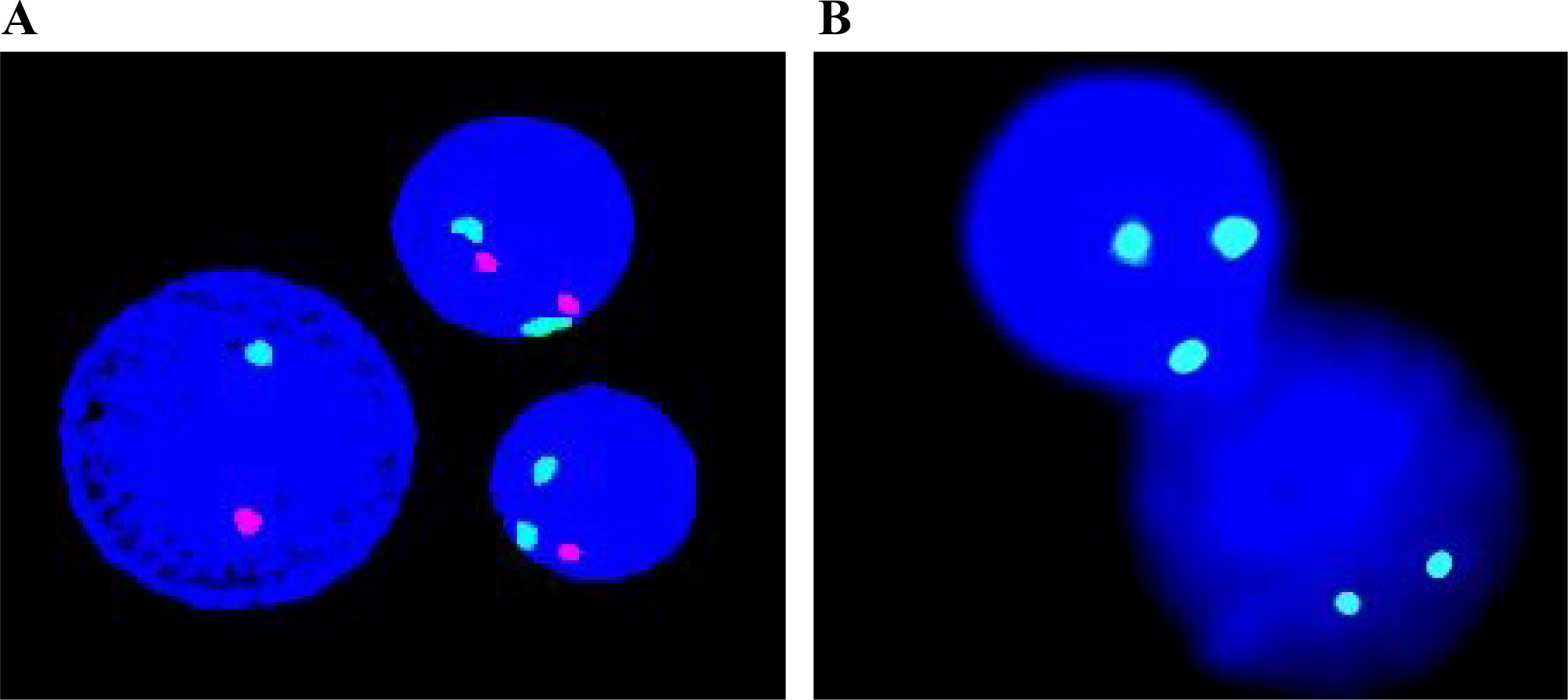
Based on individually designed probes, FISH helps to detect specific chromosomal aberrations in MDS (**A**) D7Z1/D7S486 probe indicates deletion on 7p11.1-q11.1/7q31. (**B**) D8Z2 probe reveals trisomy 8.

FISH is another important technique for molecular investigation of chromosomal alterations in MDS. FISH analysis can come up with valuable information involving the existence of small or hidden chromosomal abnormalities in patients with minor clones (Figure 2). FISH is also able to assess large numbers of interphase nuclei, so it can overcome some limitations of standard MC [87]. The diagnostic information from FISH is important for stratification of MDS into the appropriate subtypes and cytogenetic risk groups [88].

Compared with conventional chromosome banding analysis, FISH has some remarkable advantages. First, FISH can be utilized for non-proliferating cells, a large amount of cells can be assessed with relatively less lab expenditure. Second, the sensitivity of FISH is comparatively higher than traditional MC analysis, so some submicroscopic chromosomal aberrations can also be identified by FISH [89]. Third, FISH with a panel of probes can also be used to monitor disease progression and response to therapy, especially if it could be performed on peripheral blood samples [90]. Moreover, FISH can also putatively be applied to monitor lower-risk patients receiving supportive care only. The detection of cytogenetic aberrations can potentially facilitate early therapeutic interventions [91].

However, FISH should only complement MC, it will not radically substitute classical chromosomal banding analysis for an initial diagnosis of MDS. The major limitation of FISH is that it only detect particular structural or numerical chromosomal aberrations at specific locus, so those chromosomal abnormalities regarding other regions may be neglected [92, 93]. In addition, the cutoffs for positive assessment of an overwhelming majority of FISH probes are limited to approximately 5%. When metaphase cells are fewer than 20, FISH is recommended to promote the accuracy for probing recurrent MDS-related chromosome aberrations [94]. So FISH is not normally recommended for an initial screening of cytogenetic aberrations in MDS.

### SKY

Spectral karyotyping (SKY) is a novel technique for detecting chromosomal aberrations in myeloid malignancies. Based on the advancement of FISH, this technique has combined chromosome painting and multi-color fluorescence, enabling each of 23 chromosome pairs to be stained with a different color [95]. So SKY has supplemented the images of chromosomes by MC and FISH for exhibiting specific chromosomes.

The advantage of SKY is that it can unravel chromosomes of unknown origin, and clarify if one of the parents is a carrier of a balanced structural abnormality. SKY can also detect chromosomal rearrangements and minimal aberrations in MDS patients with complex karyotypes, displaying better pictures of karyotypes [96]. So SKY can overcome some defaults of the traditional banding methods, and reveal previously unrecognized chromosomal translocations. Furthermore, SKY is still crucial in detecting complex chromosomal abnormalities, so it also contributes to finding new MDS subgroups. In combination with other cytogenetic and molecular techniques, SKY may become a very powerful tool for the diagnosis, treatment and prognosis of MDS patients [97].

However, SKY can’t be used to detect structural aberrations, such as deletion, insertion, inversion, and duplication in the same clone, because these chromosomes are displayed with the same color. The resolution limit of SKY is roughly 1–2 Mb, similar to traditional chromosome banding techniques, so minor structural aberrations of less than one band cannot be visualized [98]. Therefore, SKY should combine with additional high-resolution techniques to pinpoint the site of chromosomal breakage in MDS.

### Genome-wide SNP array

The rapid progress of high-resolution genome-wide single nucleotide polymorphism-array (SNP-A) technology is characterized by hybridization of sample DNA to probes specific for allelic variants in microarrays which can detect both CNV and UPD [99]. SNP-A can precisely pinpoint the location and size of submicroscopic chromosomal aberrations. Moreover, high-resolution SNP-A has become one of the most powerful techniques to detect complex chromosomal lesions in myeloid malignancies. For instance, a recent study has utilized Affymetrix CytoScan 750K microarray to detect chromosomal loss, gain, UPD, and complex karyotypes in 162 MDS patients. Approximately 34.57% of MDS patients with complex chromosomal abnormalities were identified by CytoScan 750K microarray [100]. So SNP-A has the potential to become a very useful diagnostic technique and may complement MC and FISH in clinical cytogenetic settings.

SNP-A has many advantages over conventional techniques. First, the resolution of SNP-A is much higher than MC or FISH. Those small cryptic chromosomal loss and gain can be identified by SNP-A. Second, SNP-A doesn’t require live proliferating cells, hence it can still yield diagnostic information when routine cytogenetic methods are not feasible. Third, cryptic UPD with preserved chromosomal bandings can also be detected by SNP-A [101].

However, there are still some limitations of SNP-A. It can’t identify balanced translocations and inversions. The sensitivity of SNP-A still remains a relatively low level. The median proportion of aberrant cell clones identifiable by SNP-A is 20–30% [102].

Two factors should be considered when applying SNP-A as a clinical cytogenetic tool. First, whether SNP-A could provide additional information to routine MC and FISH. Second, whether the chromosomal aberrations detected by SNP-A have any potential clinical significance [102]. Hence combined application of SNP-A with traditional cytogenetic techniques may maximize the detection rate of chromosomal abnormalities in MDS.

### Microarray-based comparative genome hybridization (Array-CGH)

Array-CGH is an important technique for detecting CNV and UPD together in a single experiment. This method utilized competitive hybridization of differentially labeled fragmented sample DNA and control DNA to the genome at the microarray platform to detect chromosomal aberrations [103]. The fluorescence ratio of sample vs. control DNA hybridization signals is detected at different positions at the genome and yields information regarding the relative DNA copy number in the assayed genome in comparison with the normal diploid genome. Copy number alterations (CNA) of subtle chromosomal regions including potential candidate genes can be revealed [104]. The genomic resolution of the Array-CGH platform depends on the size of inserts, the space and length of DNA probes spotted on the array. So Array-CGH provides a genome-wide analysis of CNV at very high resolution. It has been reported that commercially available Array-CGH platforms have roughly 50-fold higher resolution than traditional cytogenetic methods, and can reveal chromosomal aberrations in 15% to 20% of samples [105].

The major advantage of Array-CGH over traditional cytogenetic methods is that it can detect DNA copy number alterations simultaneously at multiple loci in the genome, and can analyze a large number of genes on microarray in a single experiment [106]. Moreover, Array-CGH does not need a live, mitotically proliferating cells and can be initiated using DNA extracted from archived specimens. Analysis of Array-CGH is also objective, and feasible to automation, and can be implemented without special training or equipment [107]. However, balanced rearrangements, low-level mosaicism and polyploidy can’t be detected by Array-CGH [108].

In general, some additional cryptic chromosomal abnormalities detected by Array-CGH may improve the current diagnosis of MDS and help the assignment of appropriate phenotypes.

### Sequencing-based technologies

More recently, the progress of targeted sequencing technology has also provided valuable information for detecting chromosomal aberrations. Next-generation sequencing (NGS) technology has been utilized to detect CNV and structural variants in myeloid malignant genomes [109, 110]. These sequencing-based technologies have several advantages over conventional cytogenetic methods: (1) higher “depth” and genome-wide detection of chromosomal aberrations for the patients. (2) improved sensitivity which can detect mutations that are present in only ∼1% cells. (3) potential to monitor clonal aberrations during treatment [111]. A recent study has selectively sequenced a small portion of human genome termed Selected Target Regions (SeTRs), in order to identify genome-wide CNV, LOH and UPD. They found that SeTRs are covered by 99.73%∼99.95% with adequate depth. This new technique can identify chromosomal aberrations exempt from using a matched sample or familial information [112]. Furthermore, another study has found that NGS is highly-sensitive for accurate testing and quantification of various RUNX1 abnormalities with subsequent personalized monitoring of disease progression and therapeutic efficacy [113]. In addition, NGS can also detect chromosomal inversions and intra-chromosomal rearrangements which were not identified by SNP arrays [114]. For example, NGS data can partially simulate a large amount of breakpoints in chromosome 5. The complex structural rearrangement in chromothripsis and intra-chromosomal breakpoints were confined to a localized region of the genome [115]. So the breakpoints of deletions could be mapped with single-nucleotide resolution by NGS.

However, limitations of the sequencing-based technologies should be concerned. These methods are expensive and time-consuming, also require complicated bioinformatic analysis [116]. Owing to the inherent sequencing error rate of NGS, it is tough to reliably detect low-frequency variants [117].

Furthermore, RNA-Seq techniques can also identify novel mutation or aberrations in MDS. For instance, tRNA fragments can be accurately detected through miRNA sequencing data, the expression of these species may be useful in the diagnosis of MDS and the prediction of response to therapy [118]. Large expression differences were found for MDS-associated and novel miRNAs, which were predicted to regulate disease stage specific molecular functions and pathways, including apoptosis and response to DNA damage. Extensive post-translation editing via transfer RNAs (tRNAs) in high-grade MDS may provide a potential link for reduced apoptosis, a hallmark for this disease stage [119]. Another study has applied RNA-seq technology to study the transcriptome on 20 MDS patients and 5 age-matched controls. They identified 38 mutated genes contributing to MDS pathogenesis, including 37 genes that haven’t been reported previously. Hence RNA-seq is critical for identifying novel mutated genes in MDS. The most recurrent mutation happened in gene IFRD1 [120]. These results provide us new insights into the pathogenesis of MDS, which may inspire further investigations of diagnostic biomarkers and targeted therapies for MDS patients.

In general, sequencing-based technologies can detect a full spectrum of genomic aberrations, including single nucleotide variant (SNV), small insertion/deletion (indel), CNV, UPD, translocation, and novel mutations in MDS. The limitations of NGS have to be considered.

### Combination of multiple techniques

Given the application region, advantages and shortcomings of different techniques for detecting chromosomal aberrations in MDS, it is better to combine multiple techniques if necessary. MC in conjunction with FISH proved to be powerful to better identify additional chromosomal aberrations in MDS patients [88]. SNP-A is able to scan the whole genome and cryptic chromosomal aberrations in MDS, yet MC also can reveal balanced translocation and inversion, so the diagnostic information from MC and SNP-A are complementary, combined application of MC and SNP-A may maximize the detection rate of chromosomal abnormalities [102]. Combined aCGH with SNP-A could simultaneously detect CNV and UPD at high-resolution in a single experiment. It also provides allelic information on deletions, duplications, and amplifications [80]. CGH+SNP microarray could reveal different genetic profiles that may underlie differences in phenotypes and genetic aberrations with potential prognostic impact on MDS patients [85].

## CONCLUSIONS AND PERSPECTIVES

Although chromosomal aberrations, such as Del(5q), trisomy 8, del(20q), del(7q), monosomy 7, and complex karyotypes are prevalent in MDS, the rapid technological progress in recent years has enabled a more precise detection of multiple chromosomal abnormalities. The new findings may enhance our understanding of the molecular mechanisms underlying the pathogenesis and malignant evolution of MDS. In the future, for the multiple techniques to enter clinical application, efforts should be made to standardize the assays and refine the bioinformatic analysis for data interpretation. Further technological advance should also be made to overcome the limitations of diverse techniques.
